# Molecular Survey of Anaplasmataceae Agents, *Rickettsia* spp., *Bartonella* spp., and Piroplasmids in Ectoparasites from Cave-Dwelling Bats in Mainland Portugal

**DOI:** 10.3390/pathogens14030273

**Published:** 2025-03-12

**Authors:** Gustavo Seron Sanches, Luísa Rodrigues, Estefania Torrejón, Ricardo Bassini-Silva, Ana Cláudia Calchi, Daniel Antônio Braga Lee, Paulo Vitor Cadina Arantes, Eder Barbier, Darci Moraes Barros-Battesti, Gustavo Graciolli, Rosangela Zacarias Machado, Sandra Antunes, Ana Domingos, Marcos Rogério André

**Affiliations:** 1 Vector-Borne Bioagents Laboratory (VBBL), Departamento de Patologia, Reprodução e Saúde Única, Universidade Estadual Paulista (Unesp), Jaboticabal 14884-900, SP, Brazil; gustavoseron@hotmail.com (G.S.S.); ricardo.bassini@gmail.com (R.B.-S.); ana.calchi@unesp.br (A.C.C.); lee.danielab@gmail.com (D.A.B.L.); paulocadina@gmail.com (P.V.C.A.); barbier.eder@gmail.com (E.B.); barros.battesti@gmail.com (D.M.B.-B.); rzacariasmachado@gmail.com (R.Z.M.); 2 Instituto de Higiene e Medicina Tropical (IHMT), Universidade NOVA de Lisboa (UNL), Rua da Junqueira 100, 1349-008 Lisboa, Portugalsantunes@ihmt.unl.pt (S.A.); adomingos@ihmt.unl.pt (A.D.); 3 Instituto da Conservação da Natureza e das Florestas, Divisão de Conservação e Monitorização, 14195-165 Algés, Portugal; luisa.rodrigues@icnf.pt; 4 Laboratório de Coleções Zoológicas, Instituto Butantan, São Paulo 05503-900, SP, Brazil; 5 Laboratório de Sistemática, Ecologia e Evolução (LSEE), Instituto de Biociências, Universidade Federal de Mato Grosso do Sul, Campo Grande 79070-900, MS, Brazil; ggraciolli@yahoo.com.br; 6 Global Health and Tropical Medicine (GHTM), Associate Laboratory in Translation and Innovation Towards Global Health (LA-REAL), 1099-085 Lisbon, Portugal

**Keywords:** bat ectoparasites, *Bartonella*, Nycteribiidae, Spinturnicidae

## Abstract

Bats and their ectoparasites play a crucial role in understanding the ecology and transmission of vector-borne pathogens, yet these dynamics remain poorly studied in Portugal. This study aimed to investigate the molecular occurrence of vector-borne bacteria (*Anaplasma* spp., *Bartonella* spp., *Ehrlichia* spp., and *Rickettsia* spp.) and protozoa (*Babesia* spp. and *Theileria* spp.) in ectoparasites of cave-dwelling bats. Bats were sampled from two caves in Portugal, and their ectoparasites included wing mites (*Spinturnix myoti*), ticks (*Ixodes simplex*), and bat flies (*Penicillidia conspicua* and *Nycteribia schmidlii*). Molecular analyses revealed the presence of *Bartonella* spp. in *S. myoti* and *N. schmidlii*. Phylogenetic inference based on the *gltA* gene positioned the detected genotypes close to those previously reported in bats and Nycteribiidae flies in Europe, Asia, and Africa. Notably, no DNA from Anaplasmataceae, *Rickettsia* spp., or piroplasmids was detected. The prevalence of *S. myoti* was high, with all examined bats being infested, showing notable differences in ectoparasite diversity concerning sex and cave-specific location. These findings suggest that host behavior, environmental conditions, and ectoparasite lifecycles play critical roles in shaping pathogen transmission dynamics. This study advances the understanding of bat ectoparasite–pathogen interactions in a region with limited data and highlights the need for continued research to assess the zoonotic potential and ecological impacts of the *Bartonella* genotypes detected herein.

## 1. Introduction

The order Chiroptera comprises approximately 1487 species distributed worldwide [1]. Bats are the second most speciose group of mammals, surpassed only by rodents [2]. Among bats, 47 species are found in the European Union [3], and 27 have been recorded in mainland Portugal [4,5]. These animals are the only mammalian species capable of flying [6], making them a very diverse and widely geographically distributed group [7]. They also provide essential ecosystem services, acting as seed dispersers [8], pollinators [9], controllers of insect pests [10], and nutrient recyclers [11].

Bats are often infested by numerous ectoparasites, such as bat flies (Diptera: Nycteribiidae and Streblidae), bugs (Hemiptera: Cimicidae and Polyctenidae), fleas (Siphonaptera: Ischnopsyllidae), mites (Mesostigmata: Spinturnicidae and Macronyssidae), and ticks (Acari: Ixodidae and Argasidae) [12]. Due to most bat species’ colonial habits, which imply close contact between individuals, ectoparasite infestations can be intensified, especially in perennial shelters such as cavities [13,14].

These ectoparasites may play important roles in the transmission and maintenance of several pathogens, including some with zoonotic potential [15,16,17]. While some of them may cause mortality in bats [18], others have bats as their natural reservoirs [19,20].

Previous studies conducted in Europe have detected vector-borne agents in ectoparasites collected from bats, including piroplasmids (*Babesia* spp. and *Theileria* spp. [21,22]) and Gram-negative bacteria (*Bartonella* spp. [23,24,25], *Ehrlichia* spp. [20,22], *Anaplasma* spp. [24,26] and *Rickettsia* spp. [22,24,27,28]).

*Bartonella* spp. are the most frequently detected bacteria in bat-associated ectoparasites. In Hungary, *Bartonella* spp. have been reported in ticks (*Ixodes vespertilionis*), mites (*Steatonyssus occidentalis* and *Spinturnix myoti*), and bat flies (*Nycteribia* spp.) [23,24]. *Bartonella* DNA was detected in 28.4% of *S. myoti* mites collected from bats in Poland [24]. Szentiványi et al. [25] detected *Bartonella* spp. in *Nycteribia schmidlii* and *N. schmidlii scotti* bat flies in Spain.

A putative novel *Ehrlichia* species, namely, *Ehrlichia* sp. AvBat, was described in *Argas vespertilionis* ticks collected from bats in France [27]. Additionally, *Ehrlichia yunnan* was detected in *Ixodes simplex* ticks from bats in England [22]. *Anaplasma phagocytophilum* was molecularly detected in the blood and guano of bats in Poland [29] and France [26], respectively. *Rickettsia helvetica* was detected in *A. vespertilionis* ticks collected from bats in Hungary [28] and in *Ixodes ricinus* in Poland [23]. Additionally, *Rickettsia* spp. was identified in *A. vespertilionis* in France [20] and England [22]. More recently, [24] reported the presence of *Rickettsia* sp. in *Spinturnix myoti* collected from bats in Poland.

DNA from various Babesia species (Babesia canis, Babesia crassa, Babesia venatorum, and Babesia vesperuginis) and Theileria spp. (Theileria capreoli and Theileria orientalis) has been detected in bat-associated ticks (e.g., A. vespertilionis, Ixodes ariadnae, I. simplex, and I. vespertilionis) in Hungary, Romania [21], and England [22]. In South America, putative novel lineages of piroplasmids have been detected in both non-hematophagous bats [24] and vampire bats [14], highlighting that the diversity of piroplasmids in bats is greater than previously recognized.

This state of the art shows that studies focused on bat ectoparasites and the vector-borne agents they may carry are restricted to a few countries in Europe, and very little is known about this topic in Portugal. Therefore, the present study aimed to investigate the molecular occurrence of vector-borne bacteria (*Anaplasma* spp., *Bartonella* spp., *Ehrlichia* spp., and *Rickettsia* spp.) and protozoa (*Babesia* spp. and *Theileria* spp.) in ticks, mites, and bat flies parasitizing cave-dwelling bats from mainland Portugal.

## 2. Materials and Methods

### 2.1. Ethics Statement

Bat captures and handling followed Portuguese government laws. The Institute for Nature Conservation and Forests (ICNF) authorized the captures under permit number 81/2019/CAPT.

### 2.2. Study Area

**Figure 1:**
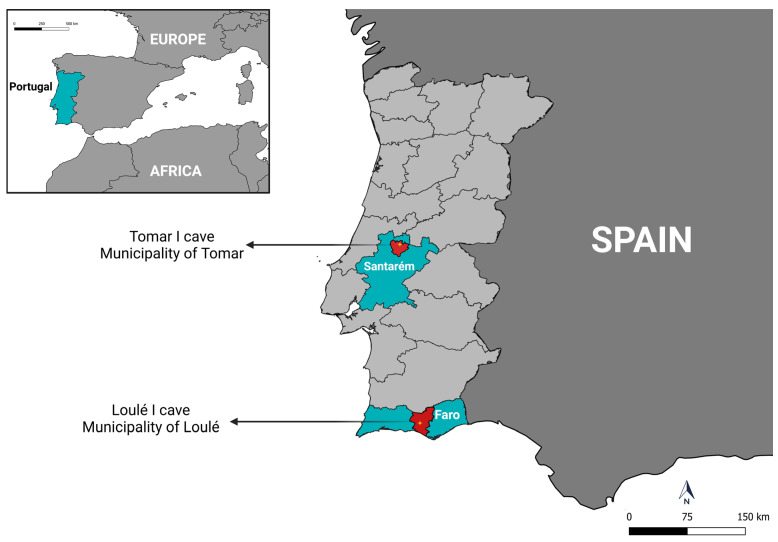
Map showing the localities of the two caves in Portugal where bats and their ectoparasites were sampled. This map was created using QGIS version 3.28.2 [https://qgis.org/ (accessed on 10 March 2025)].

This study was conducted in two roosting caves in Portugal: Cave A—Tomar I (39°39′41″ N, 8°24′57″ W), located in the municipality of Tomar, Santarém District; and Cave B—Loulé I (37°14′56″ N, 8°09′21″ W), located in the municipality of Loulé, Faro District (Figure 1). Both sites are at sea level, have a hot-summer Mediterranean climate (Köppen classification: Csa), and an annual mean temperature of 18.5 °C.

### 2.3. Bat Capture and Ectoparasite Collection

In July 2019, a single field expedition was carried out in each locality to capture bats and collect their ectoparasites. Bats were caught using harp traps at the entrance of roosting caves upon emergence, with a capture effort of 3 h per cave. During sampling, the traps were checked every 30 min, and all captured bats were stored in cloth bags. Bats were individually examined, identified at the species level and by sex [4], banded, and visually inspected for the presence of ectoparasites. Ectoparasites were removed using fine-tipped entomological forceps and stored in 1.5 mL microtubes containing absolute ethanol (Merck, Darmstadt, Germany). Bats were released immediately after sampling.

### 2.4. Ectoparasite Identification

Ticks and bat flies were carefully examined under a magnifying glass (SZX16, Olympus, Tokyo, Japan), while the wing mites were slide-mounted in Hoyer’s medium according to Walter and Krantz [30] for examination under a light microscope (BX53, Olympus, Tokyo, Japan) coupled with a digital camera (DP73, Olympus, Tokyo, Japan). Ticks were identified using the taxonomic key proposed by Hornok et al. [31]. Bat flies were identified using the taxonomic keys proposed by Theodor [32] and Mlynárová et al. [33]. In parallel, the taxonomic key proposed by Rudnick [34], as well as the original descriptions and redescriptions of each species [34,35,36], were used to identify mites.

### 2.5. DNA Extraction and Quality Assessment

DNA was extracted individually from each bat fly and tick specimen, as well as from pools comprising three wing mites collected from the same bat, using the TRIzol reagent (ThermoFisher Scientific, MA, USA), following the manufacturer’s recommendations. DNA samples were initially eluted in 100 µL of Buffer EB (Qiagen, Hilden, Germany). The DNA concentration was estimated using an ND-1000 Nanodrop Spectrophotometer (Nanodrop, Thermo Scientific, MA, USA), and the purity was checked by evaluating the absorbance ratios at 260/280 and 260/230 nm. DNA concentrations were adjusted to 50 ng/µL to serve as a template for the molecular assays. DNA samples from bat flies and wing mites were analyzed using a conventional polymerase chain reaction (cPCR) assay targeting a 710 base pair fragment of the *cox*-1 gene [37], while tick DNA samples were subjected to a PCR assay targeting a 460 base pair fragment of the 16S rRNA gene [38].

### 2.6. Molecular Screening for Vector-Borne Agents

Positive samples for the endogenous genes were subjected to specific PCR assays for Anaplasmataceae agents [39,40], *Rickettsia* spp. [41], *Bartonella* spp. [42], and piroplasmids [43], using target genes, primer sequences, and thermal cycling conditions as previously described [39,40,41,42,43]. DNA from *Anaplasma marginale* and *Babesia bovis* was used as a positive control for Anaplasmataceae and piroplasmids, respectively. For *Rickettsia* spp., DNA from *R. belli* was used as a positive control.

For Anaplasmataceae agents, *Rickettsia* spp., and piroplasmids, conventional PCR assays were performed in a T100™ Thermal Cycler (Bio-Rad™, San Diego, CA, USA) in 25 μL reaction volumes, including 12.5 μL of GoTaq^®^ Green Master Mix Reaction Buffer (pH 8.5) (Promega, WI, USA) containing 2 U/µL of GoTaq^®^ DNA Polymerase, 400 µM of dNTPs, 3 mM of MgCl2, loading dye, 1 μM of each primer, 5 μL of DNA template, and nuclease-free water to reach the final volume. Ultrapure water (Promega) was used as a negative control in all PCR assays. In the case of nested PCR, the second reaction was carried out with 1 µL of the first-round reaction product as the template. Five microliters of each amplified product from the conventional PCR assays were subjected to horizontal electrophoresis in a 1.5% agarose gel stained with 1% ethidium bromide (Life Technologies, Carlsbad, CA, USA) in 0.5x TEB run buffer (20 mM Tris, 20 mM boric acid, 0.5 mM EDTA, pH 7.2). Electrophoresis was performed at 100 V/400 W for 40 min. A DNA ladder molecular weight marker of 100 bp was used to confirm the approximate size of the amplified products. The electrophoresis gel was imaged under an ultraviolet light transilluminator ChemiDoc MP Imaging System (Bio-Rad) using Image Lab Software v4.1.

A quantitative real-time PCR (qPCR) was used to perform the screening for *Bartonella* DNA, using primers and a hydrolysis probe that target the 16S–23S rRNA intergenic transcribed spacer (ITS) region, according to Breitschwerdt et al. [42]. In duplicate, ten-microliter reactions containing 1 µL of DNA sample, 0.8 μM of each primer hydrolysis probe, Master Mix 2x buffer (GoTaq™ Probe qPCR Master Mix, Promega), and ultrapure sterilized water (Nuclease-Free Water, Promega) q.s.p. 10 μL were prepared in 96-well plates (Bio-Rad). Reactions were run on a CFX96 Connect Real-Time PCR Detection System (Bio-Rad) equipped with a FAM filter set and were further analyzed using the CFX Manager Software (Bio-Rad). An aliquot of *Bartonella henselae* DNA obtained from culture [44] was used as a positive control. Duplicates with Cq differences greater than 0.5 were repeated in triplicate. To perform the molecular characterization of *Bartonella* spp., positive samples from qPCR were subjected to cPCR assays targeting different molecular markers, namely, *gltA* (750 bp) [45], *ribC* (420 bp) [46], *rpoB* (800 bp) [47], *groEL* (752 bp) [48], *fstZ* (600 bp) [47], and *pap-31* (564 bp) [49].

### 2.7. Sequencing and Phylogenetic Analyses

PCR-amplified products were purified using the ExoSAP-IT PCR Product Cleanup Reagent (Applied Biosystems, Foster City, CA, USA) and sequenced with the BigDye Terminator v3.1 Cycle Sequencing kit (Thermo Fisher Scientific, Waltham, MA, USA) and the ABI PRISM 310 DNA Analyzer (Applied Biosystems) [50]. The obtained sequences were assembled with Sequencing Analysis 5.3.1 and submitted to BLASTn analysis [51] to infer similarities with other *Bartonella* sequences available in GenBank. Different genotypes were visually discriminated after alignment using the CLUSTAL W algorithm version 2.1 [52], which was implemented in Geneious version R11 [53].

The obtained *Bartonella* sequences were aligned with other sequences of homologous genes retrieved from GenBank using MAFFT version 7 software [available online: https://mafft.cbrc.jp/alignment/server/index.html (acessed on 10 March 2025)] [54] and edited using Bioedit v7.0.5.3 [55]. W-IQ-Tree software version 1.0 was used to select the best evolutionary model based on the Akaike Information Criterion (AIC) and to construct phylogenetic analyses using the Maximum Likelihood method [available online: http://iqtree.cibiv.univie.ac.at/ (accessed on 10 March 2025)] [56]. Clade support was evaluated through 1000 bootstrap replicates. The phylogenetic trees were edited using Treegraph 2.0.56–381 beta software [57].

### 2.8. Parasitological and Ecological Analyses

Ecological metrics and indices were calculated to compare bat communities in caves A and B: (i) abundance (total number of individuals); (ii) species richness (total number of species); (iii) Shannon’s diversity index [58]; (iv) evenness index [59]; and (v) Simpson’s diversity index [60].

Some parasitological indices were calculated to describe the association pattern of ectoparasites on host bats, in an attempt to support our understanding, for instance, of the presence, prevalence, and maintenance of possible pathogens transmitted by them. These indices are as follows: (i) prevalence; (ii) mean intensity; (iii) mean abundance; and (iv) aggregation (discrepancy index *D*) [61,62]. *D* ranges from 0 to 1, with 1 representing the maximum theoretical aggregation [62]. We calculated these indices and estimated their 95% confidence intervals (95% CI) using randomization with 2000 bootstrap replications in Quantitative Parasitology v1.0.15 [63].

We analyzed differences in ectoparasite abundance between male and female bats to explore whether host sex influenced ectoparasite load. This could directly impact the circulation of pathogens they may carry due to the biological and behavioral traits inherent to each group. These comparisons were conducted separately for each ectoparasite species, considering male and female host bats from the same cave. We applied the non-parametric Mann–Whitney *U* test to perform this analysis, as the data did not follow a normal distribution (Shapiro–Wilk test: *p* < 0.001 in all cases). Statistical significance was determined at *p* < 0.05. In order to visualize the relationships among host bats, their ectoparasites, and associated pathogens, a Sankey diagram was created. These analyses were carried out using the ‘stats’ and ‘networkD3’ packages in R v4.0.4 [64,65]. The studies were conducted on our sample’s most representative bat species, which had a balanced number of individuals across both caves.

## 3. Results

### 3.1. Bat Identification and Diversity

A total of 280 bats were collected in cave A. Of these, 270 (96.4%) were identified as belonging to the species *Miniopterus schreibersii* (Kühl, 1817) (Miniopteridae), of which 172 (63.7%) were female and 98 (36.3%) were male. The remaining ten specimens included four (1.4%) identified as males belonging to the species *Myotis myotis* (Borkhausen, 1797) (Vespertilionidae); three (1.1%) identified as females belonging to the species *Rhinolophus mehelyi* Matschie, 1901 (Rhinolophidae); two (0.7%) (one male and one female) identified as *Rhinolophus euryale* Blasius, 1853; and one (0.4%) identified as a female belonging to the species *Myotis escalerai* Cabrera, 1904.

In cave B, 110 bats were collected. Of these, 108 (98.2%) were identified as belonging to the species *M. schreibersii*, including 84 (77.8%) males and 24 (22.2%) females, and 2 (1.8%) females were identified as belonging to the species *R. mehelyi*.

Table 1 presents the abundance of bats, species richness, Shannon’s index of bat species diversity, evenness index, and Simpson’s diversity index of bats in caves A and B. It shows that cave A presented greater abundance, species richness, and diversity than cave B and that one species (*M. schreibersii*) dominates the community in both caves.

### 3.2. Ectoparasites Sampling and Identification

As a convenience sample, 42 specimens of *M. schreibersii* from each cave (29 females and 13 males from cave A, and 11 females and 31 males from cave B) were inspected for the presence of ectoparasites. Four species of ectoparasites were identified on males and females of *M. schreibersii* from both caves, totaling 679 specimens. Among these, one species of hard tick, *Ixodes simplex* Neumann, 1906 (Supplementary File), one species of wing mite, *Spinturnix myoti* (Kolenati, 1856), and two species of nycteribiid flies, *Penicillidia conspicua* Speiser, 1901, and *Nycteribia schmidlii* Schiner, 1853, were found.

On these bats, there was a 100% prevalence of the spinturnicid mite *S. myoti*. Interestingly, this mite species also exhibited the highest mean intensity and mean abundance indices, yet it had the lowest aggregation rate compared to the other ectoparasite species in the sample (Table 2). The nycteribiid fly *P. conspicua* had the second-highest prevalence, both when considering data from both caves combined and when analyzed separately by cave (Table 2). Parasitological indices for all sampled ectoparasite species are detailed in Table 2. When we compared the number of ectoparasites on bats separately by sex, only the tick *I. simplex* was significantly more abundant on female hosts in cave A (Table 3).

**Figure 2:**
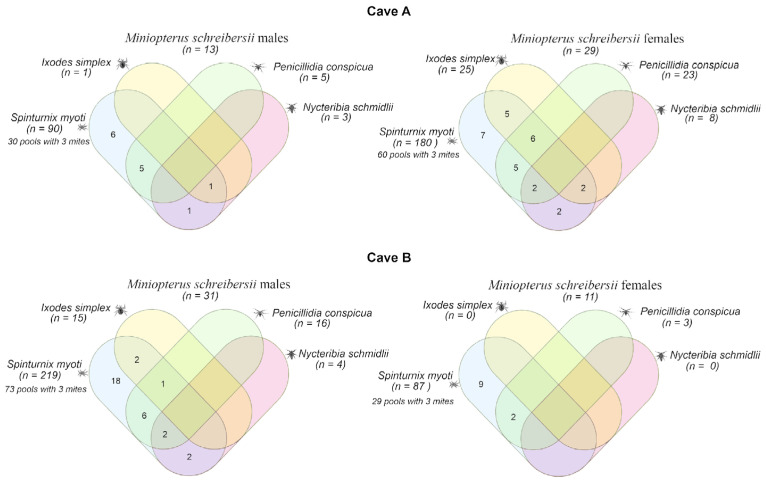
Venn diagram showing the number of male and female *Miniopterus schreibersii* from caves A and B, in Portugal, infested or coinfested with *Ixodes simplex*, *Spinturnix myoti*, *Penicillidia conspicua*, and *Nycteribia schmidlii*.

Out of the 13 *M. schreibersii* male bats sampled in cave A, 6 (46.15%) were infested only with *S. myoti*, 5 (38.46%) were coinfested with *S. myoti* and *P. conspicua*, 1 (7.69%) was coinfested with *S. myoti* and *N. schmidlii*, and 1 (7.69%) was triply infested with *I. simplex*, *S. myoti*, and *N. schmidlii*. Among the 29 females, 7 (24.13%) were infested only with *S. myoti*, 6 (20.68%) were triply infested with *S. myoti*, *I. simplex*, and *P. conspicua*, 5 (17.24%) were coinfested with *S. myoti* and *I. simplex*, 5 (17.24%) were coinfested with *S. myoti* and *P. conspicua*, and 2 (6.89%) were coinfested with *S. myoti* and *N. schmidlii*. Two (6.89%) bats were triply infested with *S. myoti*, *I. simplex*, and *N. schmidlii*, and two (6.89%) bats were triply infested with *S. myoti*, *P. conspicua*, and *N. schmidlii* (Figure 2, cave A).

Out of the 31 *M. schreibersii* male bats sampled in cave B, 18 (58.06%) were infested only with *S. myoti*, 6 (19.35%) were coinfested with *S. myoti* and *P. conspicua*, 2 (6.45%) were coinfested with *I. simplex* and *S. myoti*, 2 (6.45%) were coinfested with *S. myoti* and *N. schmidlii*, 2 (6.45%) were triply infested with *S. myoti*, *P. conspicua*, and *N. schmidlii*, and 1 (3.22%) was infested with *S. myoti*, *I. simplex*, and *P. conspicua*. Of the 11 females, 9 (81.81%) were infested only with *S. myoti*, and 2 (18.18%) were coinfested with *S. myoti* and *P. conspicua* (Figure 2, cave B). The consensus sequence confirming the taxonomic identification of ticks as *I. simplex* was deposited in the GenBank database (accession number: PQ834791).

### 3.3. Molecular Screening for Vector-Borne Pathogens

All 576 *S. myoti* pools and all individually processed bat flies (44 *P. conspicua* and 15 *N. schmidlii*) tested positive in cPCR assays targeting a 710 base pair fragment of the *cox*-1 gene. Additionally, all 41 *I. simplex* tested positive in cPCR assays targeting a 460 base pair fragment of the 16S rRNA gene. All these ectoparasite DNA samples tested negative in PCR assays for Anaplasmataceae agents, *Rickettsia* spp., and piroplasmids.

**Figure 3:**
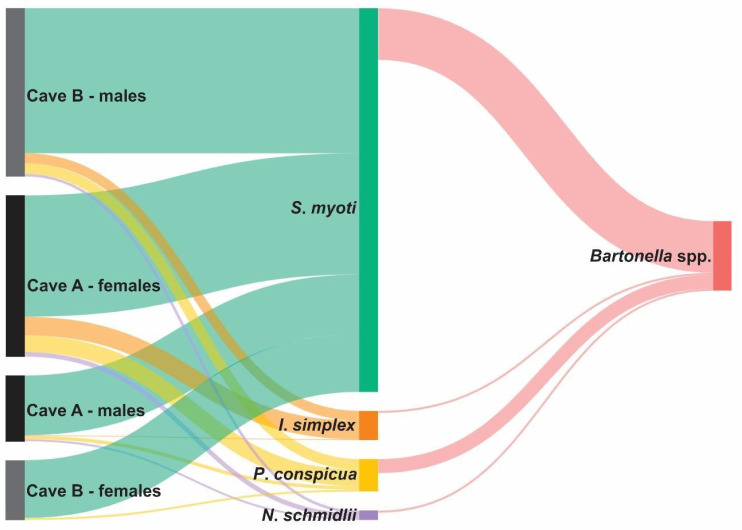
Sankey diagram based on qPCR screening results illustrating the relationships among male and female bats of the species *Miniopterus schreibersii* (left), their ectoparasites (middle), and associated *Bartonella* (right), sampled from two caves in Portugal. The thickness of the edges connecting the species represents the frequency of their associations. Pools of three mites (*S. myoti*) were used in the molecular screening for pathogens.

The screening for *Bartonella* DNA using qPCR for the 16S–23S intergenic transcribed spacer region identified 105 positive samples, of which 15/30 (50%) pools of *S. myoti* and 2/5 (40%) specimens of *P. conspicua* were obtained from males of the species *M. schreibersii* collected in cave A (Table 4); 32/61 (52.40%) pools of *S. myoti*, 11/25 (44%) specimens of *P. conspicua*, 2/7 (28.57%) specimens of *N. schmidlii*, and 2/28 (14.28%) specimens of *I. simplex* were obtained from females of the species *M. schreibersii* collected in cave A (Table 5); 22/73 (30.13%) pools of *S. myoti*, 6/16 (37.50%) specimens of *P. conspicua*, and 1/15 (2.22%) specimen of *I. simplex* were obtained from males of the species *M. schreibersii* collected in cave B (Table 6); and 8/29 (27.58%) pools of *S. myoti* and 2/3 (66.67%) specimens of *P. conspicua* were obtained from females of the species *M. schreibersii* collected in cave B (Table 7). Figure 3 illustrates the relationships between male and female bats of the species *M. schreibersii* sampled from two caves in Portugal, their ectoparasites, and their association with *Bartonella*.

### 3.4. Molecular Characterization of Bartonella spp.

Out of 105 ectoparasite samples that tested positive for *Bartonella* spp. in the qPCR targeting the 16S–23S intergenic transcribed spacer region, 15 samples (3 *P. conspicua*, 3 *N. schmidlii*, 6 *S. myoti*, and 3 *I. simplex*) with the lowest Cq values (25–26.5) were selected for direct sequencing; however, this approach yielded unreadable sequences. Consequently, six *gltA* readable sequences were obtained: one from *S. myoti* collected from female bats in cave A (GS 106); two from *N. schmidlii* collected from female bats in cave A (GS10 and GS13); and three from *S. myoti*, two of which were collected from female bats and one collected from a male bat in cave B (GS53, GS55, and GS62). Readable sequences corresponding to positive samples for the *gltA* gene are represented in Table 5, Table 6 and Table 7 with blue diamonds.

The four *Bartonella gltA* sequences from *S. myoti* were identical to each other, constituting a single genotype. On the other hand, the other two sequences (from *N. schmidlii*) corresponded to two distinct genotypes. These three sequences were deposited in the GenBank database (accession numbers: PQ835041–PQ835043).

When comparing the sequences available in GenBank, the *S. myoti*-associated *Bartonella gltA* genotype obtained herein showed 100% identity (E-value: 5 × 10^−169^; query cover: 89%) to *Bartonella* sp. (MK140192), which was detected in *P. conspicua* collected from *M. schreibersii* in Hungary.

Additionally, one of the obtained *Bartonella gltA* sequences detected in *N. schmidlii* was 100% (E-value: 0.0 to 5 × 10^−169^; query cover: 77 to 98%) identical to *Bartonella* spp. (MW007702-11, MK140343-48, LC461055, MK140254, MK140349, MK140255, MK140259-60) detected in *Miniopterus natalensis* and associated bat flies (*Nycteribia schmidlii schmidlii* and *Nycteribia schmidlii scottii*) from Romania and South Africa, respectively; in *Eptesicus serotinus*, *Miniopterus schreibersii*, and *Myotis blythii* from Georgia; and in *Eucampsipoda africana* from Zambia.

The other *gltA* sequence detected in *N. schmidlii* was 100% (E-value: 0.0 to 9 × 10^−172^; query cover: 81 to 100%) identical to *Bartonella* sp. (KT751152, KT751155, KY679154, MK140283, MK140286, MK140353-54) detected in *Miniopterus schreibersii* from Georgia; *P. conspicua* and *N. schmidlii* from Romania; and *Nycteribia stylidiopsis* from Madagascar.

### 3.5. Phylogenetic Analyses

**Figure 4:**
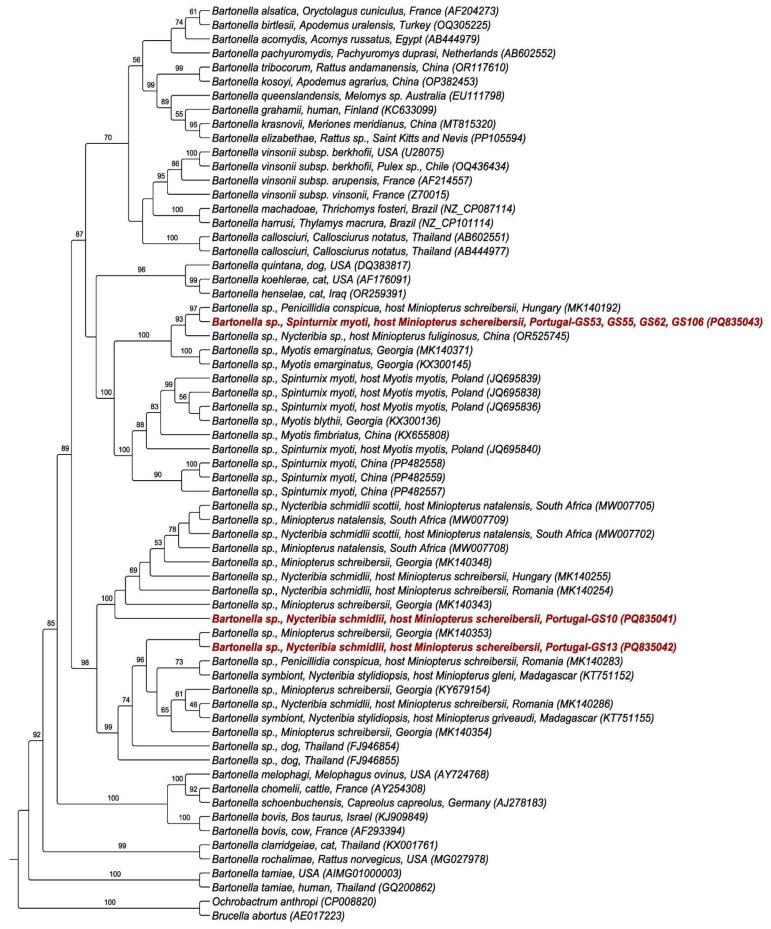
Maximum Likelihood phylogenetic inference based on a 355 bp alignment of the *Bartonella gltA* sequences and GTR + I + G evolutionary model. *Brucella abortus* and *Ochrobactrum anthropi* were used as outgroups. Numbers next to the branching points indicate the relative support from 1000 replicates.

The Maximum Likelihood (ML) phylogenetic analysis, based on a 355 bp alignment of the *gltA* gene and implemented with the GTR + I + G evolutionary model, positioned the genotype detected in *S. myoti* into a clade with other *Bartonella* sequences from bats in Europe and China, as well as their dipteran ectoparasites, with a bootstrap of 100% (Figure 4). This clade is sister to a clade containing *Bartonella* spp. sequences detected in *S. myoti* from Poland and China, as well as in bats from Georgia and China. Both clades were located close to the clade of *B. henselae*, *B. koehlerae*, and *B. quintana*. On the other hand, the sequences detected in *N. schmidlii* were positioned in two different clades, both of which are composed of *Bartonella* genotypes detected in *Miniopterus* bat species and their respective dipteran ectoparasites collected from different regions of Europe and Africa (Figure 4).

## 4. Discussion

This study provides novel insights into the molecular detection of *Bartonella* spp. in ectoparasites associated with cave-dwelling bats from Portugal, while highlighting the absence of *Ehrlichia* spp., *Anaplasma* spp., *Rickettsia* spp., and *Babesia*/*Theileria* spp. Notably, the *Bartonella* genotypes detected in *S. myoti* and *N. schmidlii* expand the known geographic and host distribution of these bacteria. Although these findings align with previous studies conducted in Europe [23,24,25,66,67], they represent the first molecular evidence of these genotypes in bat ectoparasites from Portugal, emphasizing the need for further surveys of pathogen diversity in this country. The absence of other vector-borne pathogens, such as *Rickettsia* spp., which are commonly associated with bat ectoparasites in studies from Eastern Europe (Poland and Hungary) [24,28,29], raises questions about regional or ecological factors that may influence pathogen presence in these ectoparasites.

The two obligate blood-feeding bat fly species, *Nycteribia schmidlii* and *Penicillidia conspicua*, found in our study are classified as oligoxenous since they were found on a restricted number of bat species (16 and 13, respectively), including *Miniopterus schreibersii*, which represents a quite common host–parasite interaction [28]. Interestingly, these two bat fly species are associated with *R. euryale*, *R. mehelyi*, *R. ferrumequinum*, and *M. schreibersii*, all of which share similar roosting ecology [33].

The observed difference in the abundance of *I. simplex* between male and female bats from cave A suggests that host biology, including sex-specific behaviors or physiological traits, may influence ectoparasite load and, consequently, the likelihood of pathogen transmission (e.g., [68,69,70,71,72]). In contrast, no statistically significant difference was found for other ectoparasite species or in cave B. Identifying patterns in host–parasite interactions in natural environments is inherently challenging due to the multitude of variables that may exert influence. Biological and ecological factors related to both the host (e.g., age, reproductive stage) and the parasite (e.g., developmental stage, dispersal strategy) play a crucial role [69,70,73]. Additionally, cave-specific environmental characteristics, such as microclimate and roosting density, may further shape these dynamics. Given these complexities, future studies should adopt a multifactorial approach that integrates both biotic and abiotic data to provide a more comprehensive understanding of these intricate host–parasite relationships.

*Spinturnix myoti* was the only spinturnicid mite species found among the bats sampled in this study. Indeed, the genus *Spinturnix* is the most abundant and widespread genus within the family Spinturnicidae. *Spinturnix myoti* has a broad distribution in the Palearctic region, including Portugal [74], and shows a strong parasitic preference for bats of the *Myotis* genus. Among these, *M. schreibersii* is the only bat species from the family Miniopteridae that has been recorded as a host for *S. myoti* [56], which corroborates the results found herein. Additionally, this mite species has also been found parasitizing other bat genera within the Vespertilionidae and Rhinolophidae families [74]. This finding highlights the adaptability of this mite species, demonstrating its ability to infest a variety of bat species across different families, further illustrating the ecological complexity of its parasitism. The high prevalence of *S*. *myoti* parasitizing bats in the studied caves, together with the significant association with *Bartonella* spp., in addition to the wide distribution and adaptability of this mite species to several bat species, may favor its role in the transmission of bat-associated pathogens. The real role of Spinturnicidae mites in the epidemiology of bat-associated *Bartonella* spp. should be further explored.

Ecological interactions between bats and their ectoparasites are inherently complex, with host behavior, roosting patterns, and parasite lifecycles all contributing to observed patterns of infestation and pathogen transmission. The colonial habits of *M. schreibersii* [69], which lead to high levels of social interaction and proximity, likely facilitate the spread and maintenance of ectoparasites such as *S. myoti* and *N. schmidlii*. Importantly, the focal bat species, *M. schreibersii*, is classified as Vulnerable by the International Union for Conservation of Nature (IUCN) [75] and faces additional challenges, such as habitat loss and anthropogenic pressures. This means that understanding the pathogens carried by this species can provide valuable insights into their health and survival threats.

The present study identified three *Bartonella* genotypes; while one of these included all four sequences detected in *S. myoti* obtained from bats captured in two different caves, the other two genotypes each comprised a single sequence detected in *N. schmidlii* collected from bats in the same cave. Previous studies have identified a wide variety of *Bartonella gltA* genotypes in Nycteribiidae bat flies in Europe, Asia, and Africa [72,76,77,78], which supports our findings. Despite the small number of sequences obtained from DNA samples extracted from flies, each sequence formed a distinct genotype. Strikingly, such diversity was not observed in the sequences obtained from *S. myoti*, contrary to the findings of [24], who detected three distinct *gltA* genotypes of *Bartonella* spp. in *S. myoti* collected from bats in Poland. These authors reported that genotype A consisted of 18 sequences, while the other two genotypes each contained only one sequence. The number of sequences obtained in the present study was probably not enough to reveal the full diversity of genotypes in this mite species.

The large diversity of *Bartonella* spp. circulating in bats and associated ectoparasites is clearly evidenced by phylogenetic studies, which reveal numerous clades formed by sequences detected in these animals, distributed throughout the phylogenetic tree [66,67,72,76,79,80,81,82]. In this study, the phylogenetic analysis grouped the sequences into three distinct clades, all containing sequences previously detected in bats and associated ectoparasites. The clade containing the sequence detected in *S. myoti* was a sister clade to the one containing sequences detected in the same host from Poland and China, suggesting that this might represent a novel strain circulating in these mites in Portugal.

Herein, all *I. simplex* collected from cave-dwelling bats tested negative in the qPCR for *Bartonella* spp. Previously, *Bartonella* spp. DNA was detected in 4% of *I. vespertilionis* and 11% of *I. ariadnae* collected from bats in Hungary and Romania [28]. Recently, Szentiványi et al. [82] detected *Bartonella* sp. DNA in one male specimen of *I. vespertilionis* collected in Eastern Europe. According to the authors, although the *Bartonella* DNA might represent remnant DNA from the tick’s previous blood meal on its bat host (*Rhinolophus ferrumequinum*), the vectorial competence of ticks in the transmission of *Bartonella* spp. cannot be ruled out [82].

## 5. Conclusions

This study expands our understanding of the complex interactions among bats, ectoparasites, and vector-borne bacteria. We provide the first molecular evidence of *Bartonella* spp. in bat ectoparasites from Portugal and identify *S*. *myoti* and *N*. *schmidlii* as key ectoparasites involved in the maintenance of these bacteria. Our findings also highlight the importance of host biology and environmental factors in shaping ectoparasite systems. Expanding investigations to other regions and incorporating temporal sampling will be critical to uncover the full extent of pathogen diversity and dynamics. Furthermore, assessing the zoonotic potential of bat-associated *Bartonella* genotypes and their risk of spillover to humans or domestic animals should be prioritized in future studies, given the ecological and public health implications of these findings.

## Figures and Tables

**Table 1:** Abundance (total number of individuals), species richness (total number of species), Shannon’s diversity index, Evenness index, and Simpson’s diversity index of bats recorded in caves A and B in Portugal.

	Cave A	Cave B
Abundance	280	110
Species richness	5	2
Shannon’s diversity index	0.2	0.09
Evenness index	0.124	0.131
Simpson’s diversity index	0.07	0.04

**Table 2:** Parasitological indices of ectoparasite species collected on host bats *Miniopterus schreibersii* (Chiroptera: Miniopteridae) captured in two caves in Portugal.

Ectoparasite Species	N	IH	P% (95% CI)	MI (95% CI)	MA (95% CI)	*D* (95% CI)
**Both caves**						
*Ixodes simplex*	42	17	20.2 (12.3–30.4)	2.47 (1.65–4.29)	0.500 (0.250–0.925)	0.873 (0.809–0.921)
*Penicillidia conspicua*	47	29	34.5 (24.5–45.7)	1.62 (1.31–1.97)	0.560 (0.369–0.774)	0.737 (0.650–0.810)
*Nycteribia schmidlii*	14	12	14.3 (7.6–23.6)	1.17 (1–1.33)	0.167 (0.083–0.280)	0.864 (0.773–0.918)
*Spinturnix myoti*	576	84	100 (95.7–100)	6.86 (6.21–7.54)	6.86 (6.19–7.5)	0.229 (0.193–0.272)
**Cave A**						
*Ixodes simplex*	27	14	33.3 (19.6–49.5)	1.93 (1.43–2.86)	0.643 (0.357–1.080)	0.755 (0.658–0.860)
*Penicillidia conspicua*	28	18	42.9 (27.7–59.0)	1.56 (1.22–2.00)	0.667 (0.405–0.976)	0.663 (0.552–0.787)
*Nycteribia schmidlii*	10	8	19 (8.6–34.1)	1.25 (1.00–1.25)	0.238 (0.095–0.405)	0.819 (0.694–0.899)
*Spinturnix myoti*	270	42	100 (91.6–100)	6.43 (5.64–7.14)	6.43 (5.71–7.14)	0.195 (0.153–0.246)
**Cave B**						
*Ixodes simplex*	15	3	7.1 (1.5–19.5)	5.00 (1.00–8.33)	0.357 (0.047–1.57)	0.938 (0.884–0.953)
*Penicillidia conspicua*	19	11	26.2 (13.9–42.0)	1.73 (1.18–2.36)	0.452 (0.214–0.786)	0.792 (0.700–0.877)
*Nycteribia schmidlii*	4	4	9.5 (2.7–22.6)	1 (NA)	0.095 (0.023–0.190)	0.884 (0.711–0.930)
*Spinturnix myoti*	306	42	100 (91.6–100)	7.29 (6.21–8.32)	7.290 (6.290–8.360)	0.250 (0.199–0.313)

N = number of collected ectoparasite individuals; IH = number of infested hosts; P% = prevalence in percentage; MI = mean intensity; MA = mean abundance; *D* = discrepancy index; 95% CI = 95% confidence interval using 2.000 bootstrap replications.

**Table 3:** Comparison of the number of ectoparasites (abundance) sampled on male and female *Miniopterus schreibersii* bats (Chiroptera: Miniopteridae) sampled in two caves in Portugal. Significant *p* values are in bold.

Comparison	Ectoparasite Species	Mann–Whitney *U* Test	*p*-Value
Males cave A vs. Females cave A	*Ixodes simplex*	115	**0.017**
	*Penicillidia conspicua*	159	0.375
	*Nycteribia schmidlii*	180.5	0.765
	*Spinturnix myoti*	151	0.279
Males cave B vs. Females cave B	*Ixodes simplex*	154	0.305
	*Penicillidia conspicua*	151.5	0.492
	*Nycteribia schmidlii*	148.5	0.226
	*Spinturnix myoti*	137.5	0.331

**Table 4:** Ectoparasites collected from males of the species *Miniopterus schreibersii* sampled in cave A—Tomar I (39°39′41″ N, 8°24′57″ W), located in the municipality of Tomar, Santarém District, Portugal, showing results of qPCR tests for the presence of *Bartonella* spp. DNA targeting the 16S–23S rRNA intergenic transcribed spacer. Each diamond represents one specimen of ectoparasite (for ticks and flies) or a pool with 3 specimens of mites. Black diamonds represent negative samples, and red diamonds represent positive samples for *Bartonella* spp. in qPCR; blue diamonds represent *gltA* readable sequences obtained after cPCR. F = female tick.

Single Infestation					
Species	ID	*I. simplex*	*P. conspicua*	*N. schmidlii*	*S. myoti* (Pools of 3)
*Miniopterus schreibersii*	23				♦♦
*Miniopterus schreibersii*	3				♦♦♦
*Miniopterus schreibersii*	28				♦♦♦
*Miniopterus schreibersii*	36				♦♦♦
*Miniopterus schreibersii*	29				♦♦
*Miniopterus schreibersii*	41				♦♦
**Double infestation**					
*Miniopterus schreibersii*	39		♦		♦♦
*Miniopterus schreibersii*	8		♦		♦♦♦
*Miniopterus schreibersii*	16		♦		♦♦
*Miniopterus schreibersii*	10		**♦**		♦♦
*Miniopterus schreibersii*	42		**♦**		♦♦
*Miniopterus schreibersii*	33			♦	♦
**Triple infestation**					
*Miniopterus schreibersii*	13	F:♦		♦♦	♦♦♦

**Table 5:** Ectoparasites collected from females of the species *Miniopterus schreibersii* sampled in cave A—Tomar I (39°39′41″ N, 8°24′57″ W), located in the municipality of Tomar, Santarém District, Portugal, showing results of qPCR tests for the presence of *Bartonella* spp. DNA targeting the 16S–23S rRNA intergenic transcribed spacer. Each diamond represents one specimen of ectoparasite (for ticks and flies) or a pool with 3 specimens of mites. Black diamonds represent negative samples, and red diamonds represent positive samples for *Bartonella* spp. in qPCR; blue diamonds represent *gltA* readable sequences obtained after cPCR. L = larva; N = nymph; F = female tick.

Single Infestation					
Species	ID	*I. simplex*	*P. conspicua*	*N. schmidlii*	*S. myoti* (Pools of 3)
*Miniopterus schreibersii*	1				♦
*Miniopterus schreibersii*	2				♦
*Miniopterus schreibersii*	40				♦♦
*Miniopterus schreibersii*	21				♦♦
*Miniopterus schreibersii*	37				♦♦♦
*Miniopterus schreibersii*	35				♦♦ ♦
*Miniopterus schreibersii*	17				♦♦♦
**Double infestation**					
*Miniopterus schreibersii*	24		**♦**		♦
*Miniopterus schreibersii*	32		**♦**		♦♦
*Miniopterus schreibersii*	31		♦♦		♦♦
*Miniopterus schreibersii*	19		♦		♦♦♦
*Miniopterus schreibersii*	22		**♦♦**		♦♦
*Miniopterus schreibersii*	30			**♦**	♦♦♦♦
*Miniopterus schreibersii*	5			♦	♦♦
*Miniopterus schreibersii*	9	F: ♦♦♦♦♦♦			♦
*Miniopterus schreibersii*	11	N: ♦			♦♦
*Miniopterus schreibersii*	4	F: ♦			♦♦
*Miniopterus schreibersii*	6	F: ♦♦			♦♦♦
*Miniopterus schreibersii*	18	F: ♦			♦♦♦♦
**Triple infestation**					
*Miniopterus schreibersii*	15	L: ♦ N: ♦	♦♦		♦
*Miniopterus schreibersii*	20	F: ♦ N: ♦	♦		♦
*Miniopterus schreibersii*	25	F: ♦ N: ♦ L: ♦	**♦♦**		♦
*Miniopterus schreibersii*	34	F: ♦	**♦♦**		♦♦
*Miniopterus schreibersii*	25	F: ♦ N: ♦ L: ♦	**♦♦**		♦
*Miniopterus schreibersii*	12	F: ♦ N: ♦	**♦♦♦♦**		♦♦
*Miniopterus schreibersii*	27	F: ♦	**♦♦♦**		♦♦
*Miniopterus schreibersii*	7		♦	♦	♦♦
*Miniopterus schreibersii*	38		**♦**	**♦**	♦♦♦
*Miniopterus schreibersii*	14	N: ♦		♦	♦
*Miniopterus schreibersii*	26	N: ♦♦		**♦♦**	♦♦

**Table 6:** Ectoparasites collected from males of the species *Miniopterus schreibersii* sampled in cave B—Loulé I (37°14′56″ N, 8°09′21″ W), located in the municipality of Loulé, Faro District, Portugal, showing results of qPCR tests for the presence of *Bartonella* spp. DNA targeting the 16S–23S rRNA intergenic transcribed spacer. Each diamond represents one specimen of ectoparasite (for ticks and flies) or a pool with 3 specimens of mites. Black diamonds represent negative samples, and red diamonds represent positive samples for *Bartonella* spp. in qPCR; blue diamonds represent *gltA* readable sequences obtained after cPCR. L = larva; N = nymph; F = female tick.

Single Infestation					
Species	ID	*I. simplex*	*P. conspicua*	*N. schmidlii*	*S. myoti* (Pools of 3)
*Miniopterus schreibersii*	31				♦
*Miniopterus schreibersii*	35				♦
*Miniopterus schreibersii*	28				♦
*Miniopterus schreibersii*	26				♦♦
*Miniopterus schreibersii*	18				♦♦
*Miniopterus schreibersii*	5				♦♦
*Miniopterus schreibersii*	41				♦♦♦
*Miniopterus schreibersii*	25				♦
*Miniopterus schreibersii*	19				♦♦
*Miniopterus schreibersii*	29				♦♦
*Miniopterus schreibersii*	36				♦♦♦
*Miniopterus schreibersii*	13				♦♦♦
*Miniopterus schreibersii*	8				♦♦♦
*Miniopterus schreibersii*	4				♦♦♦
*Miniopterus schreibersii*	6				♦♦♦
*Miniopterus schreibersii*	1				♦♦♦♦♦
*Miniopterus schreibersii*	2				♦♦♦♦♦
*Miniopterus schreibersii*	3				♦♦♦♦♦♦
**Double infestation**					
*Miniopterus schreibersii*	14		♦		♦
*Miniopterus schreibersii*	11		♦		♦♦
*Miniopterus schreibersii*	32		♦♦♦		♦♦
*Miniopterus schreibersii*	10		♦♦♦♦		♦♦
*Miniopterus schreibersii*	39		♦		♦♦
*Miniopterus schreibersii*	40		♦		♦♦
*Miniopterus schreibersii*	42	N: ♦♦♦♦♦♦♦ L: ♦♦♦♦			♦♦
*Miniopterus schreibersii*	12	N: ♦			♦♦
*Miniopterus schreibersii*	17			♦	♦
*Miniopterus schreibersii*	38			♦	♦♦♦
**Triple infestation**					
*Miniopterus schreibersii*	24		♦	♦	♦♦
*Miniopterus schreibersii*	30		♦♦	♦	♦♦♦
*Miniopterus schreibersii*	20	F: ♦♦ N: ♦	♦♦		♦

**Table 7:** Ectoparasites collected from females of the species *Miniopterus schreibersii* sampled in cave B—Loulé I (37°14′56″ N, 8°09′21″ W), located in the municipality of Loulé, Faro District, Portugal, showing results of qPCR tests for the presence of *Bartonella* spp. DNA targeting the 16S–23S rRNA intergenic transcribed spacer. Each diamond represents one specimen of ectoparasite (for ticks and flies) or a pool with 3 specimens of mites. Black diamonds represent negative samples, and red diamonds represent positive samples for *Bartonella* spp. in qPCR; blue diamonds represent *gltA* readable sequences obtained after cPCR. L = larva; N = nymph; F = female tick.

Single Infestation					
Species	ID	*I. simplex*	*P. conspicua*	*N. schmidlii*	*S. myoti* (Pools of 3)
*Miniopterus schreibersii*	27				♦
*Miniopterus schreibersii*	7				♦♦
*Miniopterus schreibersii*	34				♦♦♦
*Miniopterus schreibersii*	21				♦♦♦
*Miniopterus schreibersii*	37				♦♦♦♦
*Miniopterus schreibersii*	23				♦♦
*Miniopterus schreibersii*	16				♦♦
*Miniopterus schreibersii*	22				♦♦♦♦
*Miniopterus schreibersii*	33				♦♦♦♦
**Double infestation**					
Miniopterus schreibersii	9		♦		♦♦♦
Miniopterus schreibersii	15		♦♦		♦

## Data Availability

The datasets supporting the conclusions of this article are included within the article.

## Supplementary Materials

The following supporting information can be downloaded at: https://www.mdpi.com/article/10.3390/pathogens14030273/s1, Figure S1: Light micrograph of an *Ixodes simplex* female tick under a magnifying glass (SZX16, Olympus, Tokyo, Japan) coupled with a digital camera (DP73, Olympus, Tokyo, Japan). Magnification = N x.
