# Supplementary figures and images for: Molecular Survey of Anaplasmataceae Agents, Rickettsia spp., Bartonella spp., and Piroplasmids in Ectoparasites from Cave-Dwelling Bats in Mainland Portugal
Source: Pathogens. 2025 Mar 12;14(3):273. doi: 10.3390/pathogens14030273 (PMC11946244; doi:10.3390/pathogens14030273)

A

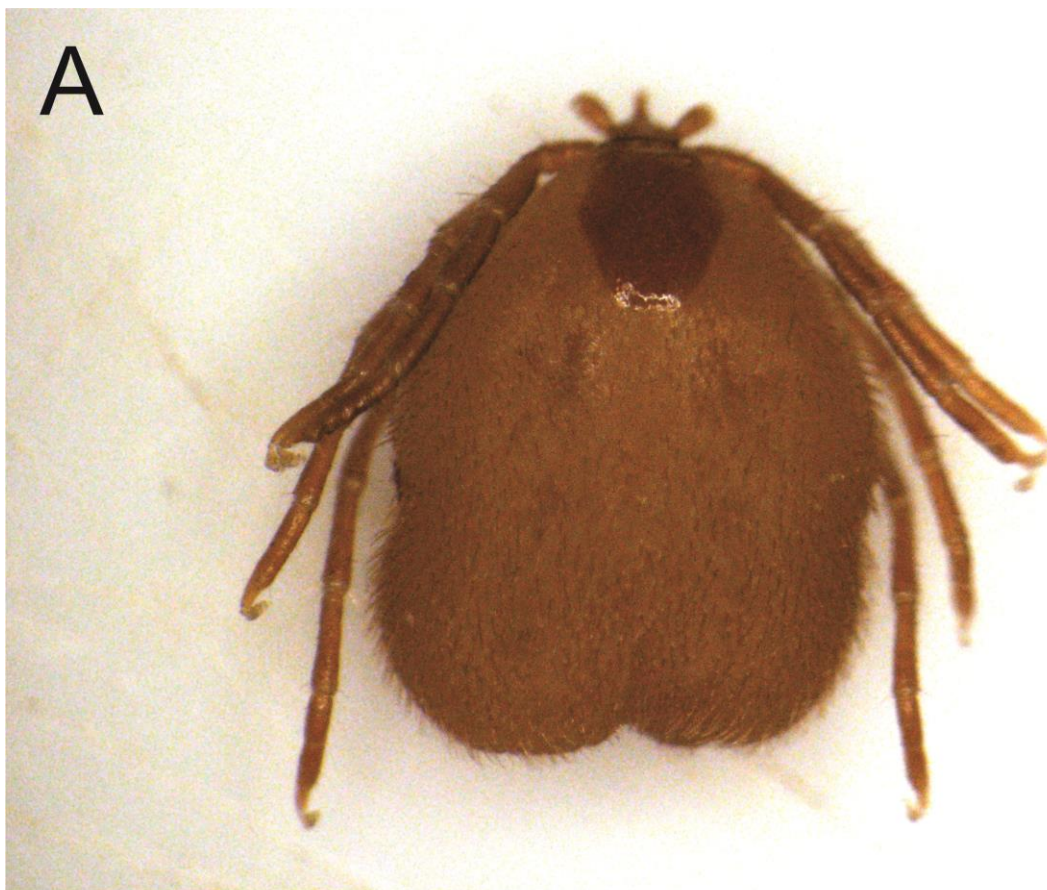

B

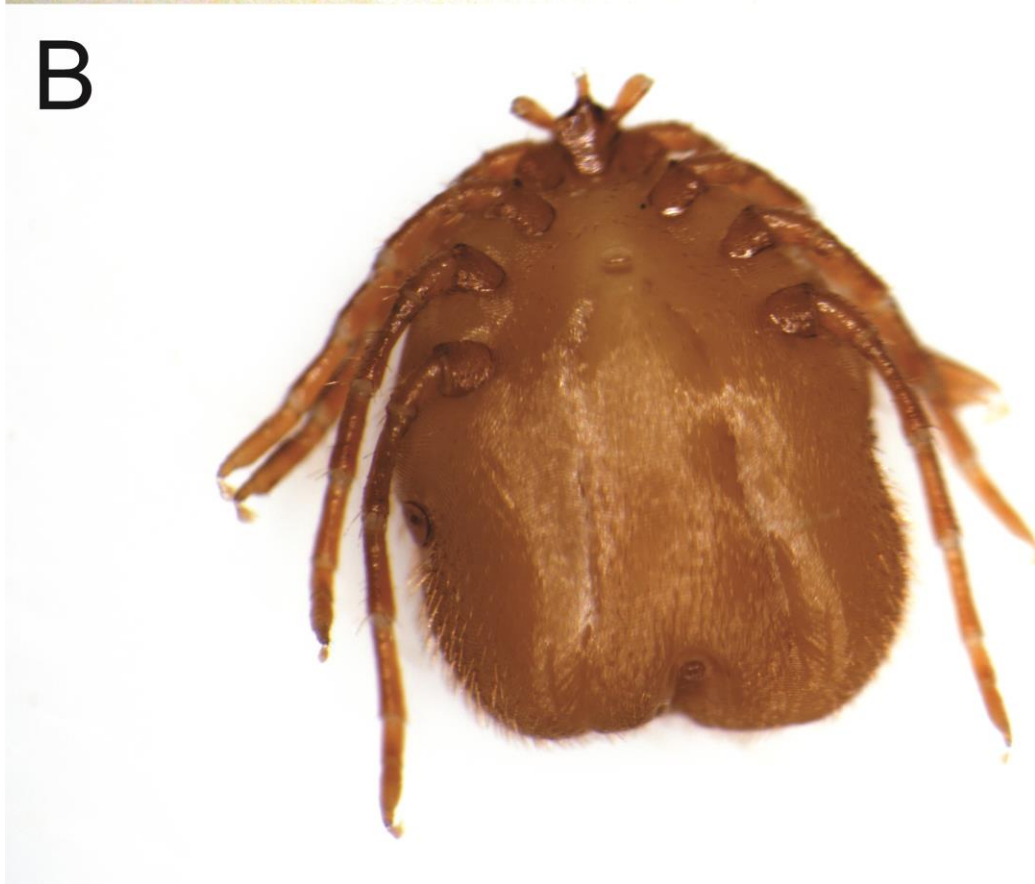

Supplement: Supplementary file 1 [file pathogens-14-00273-s001.zip › pathogens-3471326-supplementary.pdf]
